# BRG1 Stimulates Endothelial Derived Alarmin MRP8 to Promote Macrophage Infiltration in an Animal Model of Cardiac Hypertrophy

**DOI:** 10.3389/fcell.2020.00569

**Published:** 2020-07-07

**Authors:** Zilong Li, Yuanyuan Zhang, Yangxi Zhang, Liming Yu, Bin Xiao, Tianfa Li, Xiaocen Kong, Yong Xu

**Affiliations:** ^1^Pancreas Center, First Affiliated Hospital of Nanjing Medical University, Nanjing, China; ^2^Key Laboratory of Targeted Intervention of Cardiovascular Disease and Collaborative Innovation Center for Cardiovascular Disease, Department of Pathophysiology, Nanjing Medical University, Nanjing, China; ^3^Institute of Biomedical Research, Liaocheng University, Liaocheng, China; ^4^Department of Cardiovascular Medicine, Affiliated Hospital of Hainan Medical University, Haikou, China; ^5^Department of Endocrinology, Nanjing First Hospital, Nanjing Medial University, Nanjing, China

**Keywords:** transcriptional regulation, macrophage infiltration, endothelial cell, Angiotensin II, cardiac hypertrophy

## Abstract

Endothelial cell derived angiocrine factors contribute to the disruption of homeostasis and the pathogenesis of cardiovascular diseases in response to stress stimuli. In the present study we investigated the role of BRG1, a key component of the chromatin remodeling complex, in the regulation of angiocrine signaling. We report that angiotensin II (Ang II) induced pathological cardiac hypertrophy was attenuated in mice with endothelial-specific ablation of BRG1 (ecKO) compared to the control mice (WT). Mitigation of cardiac hypertrophy as a result of BRG1 deficiency was accompanied by decreased macrophage homing to the hearts. This could be explained by the observation that the ecKO mice exhibited down-regulation of myeloid-related protein 8 (MRP8), a well-established chemokine for macrophages, in vascular endothelial cells compared to the WT mice. Further analysis revealed that BRG1 mediated the activation of MRP8 expression by Ang II treatment in endothelial cells to promote macrophage migration. BRG1 was recruited to the MRP8 promoter by interacting with hypoxia-inducible factor 1 (HIF-1α). Reciprocally, BRG1 facilitated the binding of HIF-1α to the MRP8 promoter by sequentially recruiting histone acetyltransferase p300 and histone demethylase KDM3A. Depletion of either p300 or KDM3A repressed the induction of MRP8 expression by Ang II and ameliorated macrophage migration. In conclusion, our data delineate a novel epigenetic pathway whereby Ang II stimulates MRP8 production and macrophage homing to promote cardiac hypertrophy.

## Introduction

Inflammation is considered a double-edged sword that plays key roles both maintaining and disrupting the internal homeostasis (Medzhitov, 2008). Macrophages represent a heterogeneous population of cells that contribute to the modulation of inflammatory response (Kim et al., 2015). Based on origins, macrophages can be roughly classified into two categories: those derived from circulation and those native to specific tissue or organ (Honold and Nahrendorf, 2018). In order for blood-borne macrophages to navigate to their destinations and exert the pro-inflammatory effects, chemoattractive cues need to be produced and released into the circulation in the event of various pathophysiological processes. Macrophage recruitment is both a hallmark event and a pathogenic factor in the development of a myriad of cardiovascular diseases. For instance, during the pathogenesis of cardiac hypertrophy and heart failure, there is increased macrophage infiltration in the myocardium where they aid the activation and differentiation of lymphocytes to perpetuate the inflammatory response and dampen heart function (Bansal et al., 2017; Patel et al., 2018). In accordance, ample evidence suggests that chemical depletion (via clodronate) or genetic deletion (via the diphtheria toxin/DT receptor system) of macrophage lineages in mice is associated with attenuated cardiac inflammation and improvement of heart function (Chen and Frangogiannis, 2018).

Angiotensin II (Ang II) is a pleiotropic hormone playing key roles in the pathogenesis of cardiovascular diseases. There is compelling evidence that elevation of plasma Ang II levels is associated with pathological cardiac hypertrophy and heart failure in humans (Roig et al., 2000; van de Wal et al., 2006) and in model animals (Ai et al., 2009; Zhang et al., 2017). The assertion that Ang II is essential for the pathogenesis of cardiac hypertrophy is further buttressed by the observations that either blockade of its synthesis (by the angiotensin II converting enzyme inhibitors) or disruption of its signaling cascade (by genetic deletion of its receptor/AT_2_R or by administration of AT_2_R antagonists) is cardioprotective in rodents and in patients with heart failure (Opie and Sack, 2001). Of interest, cardiac hypertrophy induced by enhanced Ang II activity is accompanied by augmented macrophage infiltration in the myocardium (Ichihara et al., 2001; Wu et al., 2002; Sopel et al., 2011). In addition, Ang II has been shown to stimulate the expression of a host of chemoattractive molecules, including MCP-1 (Matsuda et al., 2015), CXCL1 (Wang L. et al., 2018), and IL-8 (Schmeisser et al., 2004), to promote macrophage migration both *in vitro* and *in vivo*; the underlying epigenetic mechanism whereby Ang II activates the transcription of chemokines is not completely understood.

Gene expression in mammalian cells is intimately influenced by the epigenetic machinery consisting of histone/DNA modifying enzymes, non-regulatory RNAs, and chromatin remodeling proteins (Ledford, 2015). Brahma related gene 1 (BRG1) is a component of the SWI/SNF chromatin remodeling complex providing the ATPase activity to mobilize nucleosomes and shaping up the chromatin landscape (Xu and Fang, 2012). In the cardiovascular system, it has been found that constitutive deletion of BRG1 in endothelial cells causes defective capillary network formation and premature death whereas induced deletion of BRG1 in the same compartment post-birth is tolerable with no overt phenotype in mice (Griffin et al., 2011; Wiley et al., 2015). A string of recent reports suggest that BRG1 can modulate specific transcription events in endothelial cells to promote the pathogenesis of atherosclerosis (Fang et al., 2013), pulmonary hypertension (Chen et al., 2013), cardiac ischemia-reperfusion injury (Zhang et al., 2018c), and abdominal aortic aneurysm (Zhang et al., 2018b); in each of these cases, BRG1 deficiency in endothelial cells is invariably mirrored by compromised recruitment of circulating immune cells. Here we report that BRG1 stimulates endothelial derived alarmin MRP8 to promote macrophage infiltration and cardiac hypertrophy in mice. Therefore, endothelial BRG1 may be considered as a desirable target for the intervention of inflammation-associated cardiovascular diseases.

## Materials and Methods

### Animals

All the animal experiments were reviewed and approved by the intramural Ethics Committee on Humane Treatment of Experimental Animals. Endothelial-specific deletion of BRG1 was achieved by crossing the *Smarca4*^f/f^ strain (Li et al., 2018a, b) with the *Cdh5*-Cre strain (Li et al., 2018e). Male, 8-week old mice were induced to develop cardiac fibrosis by Angiotensin II (1 μg/kg/min) infusion for two consecutive weeks using subcutaneously implanted minipumps (Alzet 2002). Cardiac hypertrophy was monitored by echocardiography (GE Vivid 7 equipped with a 14-MHz phase array linear transducer, S12, allowing a 150 maximal sweep rate).

### Cell Culture, Plasmids, and Transient Transfection

Immortalized human endothelial cells (EAhy926, ATCC), mouse macrophage-like cells (RAW264.7, ATCC), and HEK293 cells were maintained in DMEM supplemented with 10% fetal bovine serum (FBS, Hyclone). Angiotensin II was purchased from Sigma. MRP8 promoter-luciferase constructs (Grebhardt et al., 2012) and BRG1 expression constructs (Li et al., 2019a) have been previously described. Small interfering RNAs were purchased from Dharmacon. PFI-3 was purchased from Selleck. Transient transfections were performed with Lipofectamine 2000. Luciferase activities were assayed 24–48 h after transfection using a luciferase reporter assay system (Promega) as previously described (Li et al., 2019c). For conditioned media (CM) collection, the cells were switched to and incubated with serum-free media overnight. The next day, the media were collected, centrifuged at 4000 × *g* for 30 min at 4°C using 3-kDa MW cut-off filter units (Millipore) and sterilized through a 0.4-μm filter.

### Protein Extraction and Western Blot

Whole cell lysates were obtained by re-suspending cell pellets in RIPA buffer (50 mM Tris pH7.4, 150 mM NaCl, 1% Triton X-100) with freshly added protease inhibitor (Roche) as previously described (Li et al., 2018c, d; Liu et al., 2018). Western blot analyses were performed with anti-BRG1 (Santa Cruz, sc-10768), anti-MRP8 (Proteintech, 15792-1-AP), anti-HIF-1α (Santa Cruz, sc-10790), anti-p300 (Santa Cruz, sc-585), anti-KDM3A (Proteintech, 15792-1-AP), and anti-β-actin (Sigma, A2228) antibodies. For densitometrical quantification, densities of target proteins were normalized to those of β-actin. Data are expressed as relative protein levels compared to the control group which is arbitrarily set as 1.

### RNA Isolation and Real-Time PCR

RNA was extracted with the RNeasy RNA isolation kit (Qiagen). For cardiac tissue homogenization, the heart was dissected and cut into small pieces (∼20 mg) using a sterilized blade. The appropriately sized cardiac tissue was placed into a microcentrifuge tube along with stainless steel beads (1.6 mm) and 350 μl lysis buffer. The tubes were placed in the Bullet Blender^TM^ (Scientific Instrument Services) for homogenization. The homogenized tissue lysates were used for RNA extraction per manual instruction. Reverse transcriptase reactions were performed using a SuperScript First-strand Synthesis System (Invitrogen) as previously described (Li et al., 2018a, b; Liu et al., 2018). Real-time PCR reactions were performed on an ABI Prism 7500 system with the following primers: *MRP8*, 5′-AATTTCCA TGCCGTCTACAG-3′ and 5′-CGCCCATCTTTATCACCAG-3′; *BRG1*, 5′-TCATGTTGGCGAGCTATTTCC-3′ and 5′-GGTTCC GAAGTCTCAACGATG-3′; *HIF1A*, 5′-TTCACCTGAGCCTAA TAGTCC-3′ and 5′-CAAGTCTAAATCTGTGTCCTG-3′; *p300*, 5′-GCGGCCTAAACTCTCATCT-3′ and 5′-TCTGGTAAGTCG TGCTCCAA-3′; *KDM3A*, 5′-TTCTTTTCCTCCAAGATTC CC-3′ and 5′-GGGACCATTCGAGCTGTTT-3′. Ct values of target genes were normalized to the Ct values of housekeekping control gene (18 s, 5′-CGCGGTTCTATTTTGTTGGT-3′ and 5′-TCGTCTTCGAAACTCCGACT-3′ for both human and mouse genes) using the ΔΔCt method and expressed as relative mRNA expression levels compared to the control group which is arbitrarily set as 1.

### Chromatin Immunoprecipitation

Chromatin immunoprecipitation (ChIP) assays were performed essentially as described before (Li et al., 2019b). In brief, chromatin in control and treated cells were cross-linked with 1% formaldehyde. Cells were incubated in lysis buffer (150 mM NaCl, 25 mM Tris pH 7.5, 1% Triton X-100, 0.1% SDS, 0.5% deoxycholate) supplemented with protease inhibitor tablet and PMSF. DNA was fragmented into ∼200 bp pieces using a Branson 250 sonicator. Aliquots of lysates containing 200 μg of protein were used for each immunoprecipitation reaction with anti-BRG1 (Santa Cruz, sc-10768), anti-anti-acetyl H3 (Millipore, 06-599), anti-dimethyl H3K9 (Millipore, 17-648), anti-HIF-1α (Santa Cruz, sc-10790), anti-p300 (Santa Cruz, sc-585), anti-KDM3A (Abcam, ab-91252), or pre-immune IgG. For re-ChIP, immune complexes were eluted with the elution buffer (1% SDS, 100 mM NaCO_3_), diluted with the re-ChIP buffer (1% Triton X-100, 2 mM EDTA, 150 mM NaCl, 20 mM Tris pH 8.1), and subject to immunoprecipitation with a second antibody of interest.

### Macrophage Migration Assay

Macrophage migration was measured using the Boyden chamber inserts (5 μm, Corning) as previously described (Zhang et al., 2018b). Briefly, RAW264.7 cells were added to the upper chamber whereas the conditioned media collected from endothelial cells were added to the lower chamber. The number of migrated macrophages in the lower chamber was counted in five randomly chosen fields using an inverted microscope. In certain experiments, recombinant human MRP8 (20 ng/ml, R&D) was directly added to the conditioned media. Migrated macrophages were counted in at least five different fields for each well. All experiments were performed in triplicates and repeated three times.

### Immunofluorescence Staining

For immunofluorescence staining, paraffin sections (5 μm) of aortic arteries were permeabilized with 0.1% Triton X-100 in PBS for 10 min and then blocked with 5% BSA for 20 min at room temperature followed by incubating with anti-CD31 (Abcam, ab28364) and anti-MRP8 (Abcam, ab17050) overnight at 4°C. The nuclei were counterstained with DAPI (Sigma).

### Statistical Analysis

Data are presented as mean ± SD. Data normality was examined by the Shapiro–Wilk test. For normally distributed data, comparisons between two groups were performed using Student’s *t*-test, and comparisons among multiple groups were performed using ANOVA followed by *post hoc* Bonferroni correction. For non-normal data comparisons were performed by the Mann–Whitney U test or the Kruskal–Wallis test followed by Dunn’s multiple comparison test. Data homoscedasticity was examined by the Bartlett’s and Brown–Forsythe test. Non-homoscedastic data was analyzed by the Brown-Forsythe ANOVA test followed by Dunnett’s T3 multiple comparisons test. A *p*-value < 0.05 was considered significant.

## Results

### Endothelial-Specific BRG1 Deletion in Mice Attenuates Ang II Infusion Induced Macrophage Infiltration in the Hearts

**FIGURE 1:**
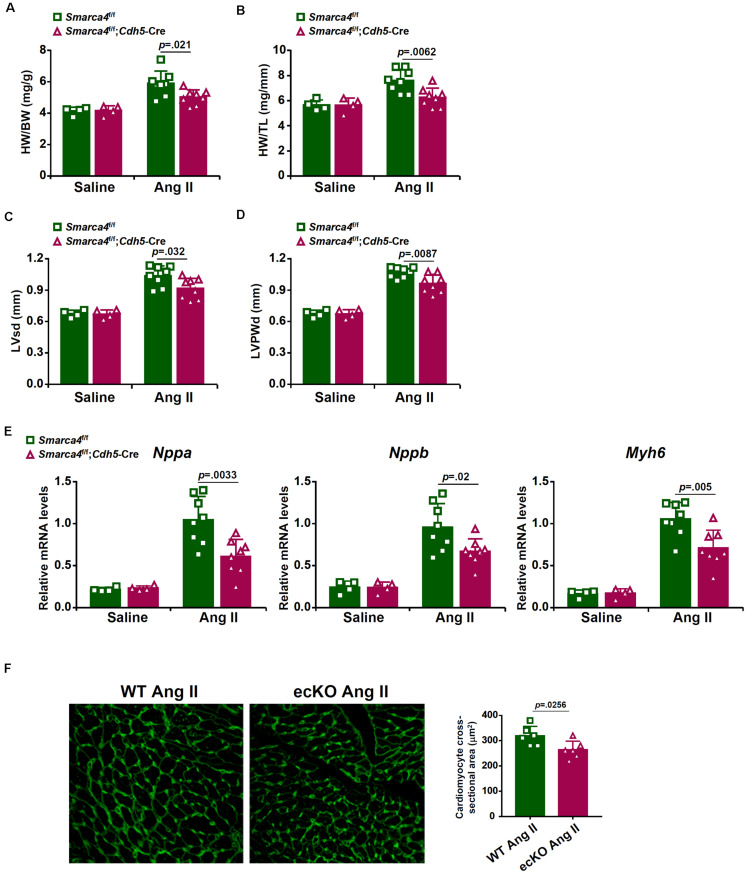
Endothelial-specific BRG1 deletion in mice attenuates Ang II infusion induced cardiac hypertrophy in the hearts. Wild type (*Smarca4*^f/f^) and endothelial BRG1 knockout (*Smarca4*^f/f^; *Cdh5*-Cre) mice were implanted with Ang II minipumps to induce cardiac hypertrophy as described in Methods. **(A)** Heart weight/body weight ratios. **(B)** Heart weight/tibia bone length ratios. **(C)** Left ventricular end-systolic dimension values were measured by Doppler echocardiography. **(D)** Left ventricular posterior wall dimension values were measured by Doppler echocardiography. **(E)** Expression levels of hypertrophic genes were examined by qPCR. **(F)** Paraffin sections were stained with WGA and cardiomyocyte size was calculated by Image Pro. *N* = 4 mice for the saline groups and *N* = 8 mice for the Ang II groups.

We first compared the phenotype of the mice with a deficiency of BRG1 in endothelial cells (*Smarca4*^f/f^; *Cdh5*-Cre, referred to as ecKO) and the control mice (*Smarca4*^f/f^, referred to as WT) in response to chronic Ang II infusion. Ang II infusion induced a robust pro-hypertrophic response in the heart as evidenced by the elevation of heart weight/body weight ratio (Figure 1A), heart weight/tibia bone length ratio (Figure 1B), left ventricular systolic dimension (Figure 1C), and left ventricular posterior wall dimension (Figure 1D). For each one of these measurements, it was observed that endothelial specific BRG1 deletion attenuated the pro-hypertrophic response. Quantitative PCR analysis of message levels of pro-hypertrophic genes, including *Nppa*, *Nppb*, and *Myh6*, confirmed that cardiac hypertrophy was ameliorated in the ecKO mice compared to the WT mice (Figure 1E). In addition, WGA staining also showed that cross-sectional areas of cardiomyocytes were smaller in the ecKO hearts than the WT hearts indicative of a reduced pro-hypertrophic response (Figure 1F).

### BRG1 Regulates Endothelium-Derived MRP8 in Mice

**FIGURE 2:**
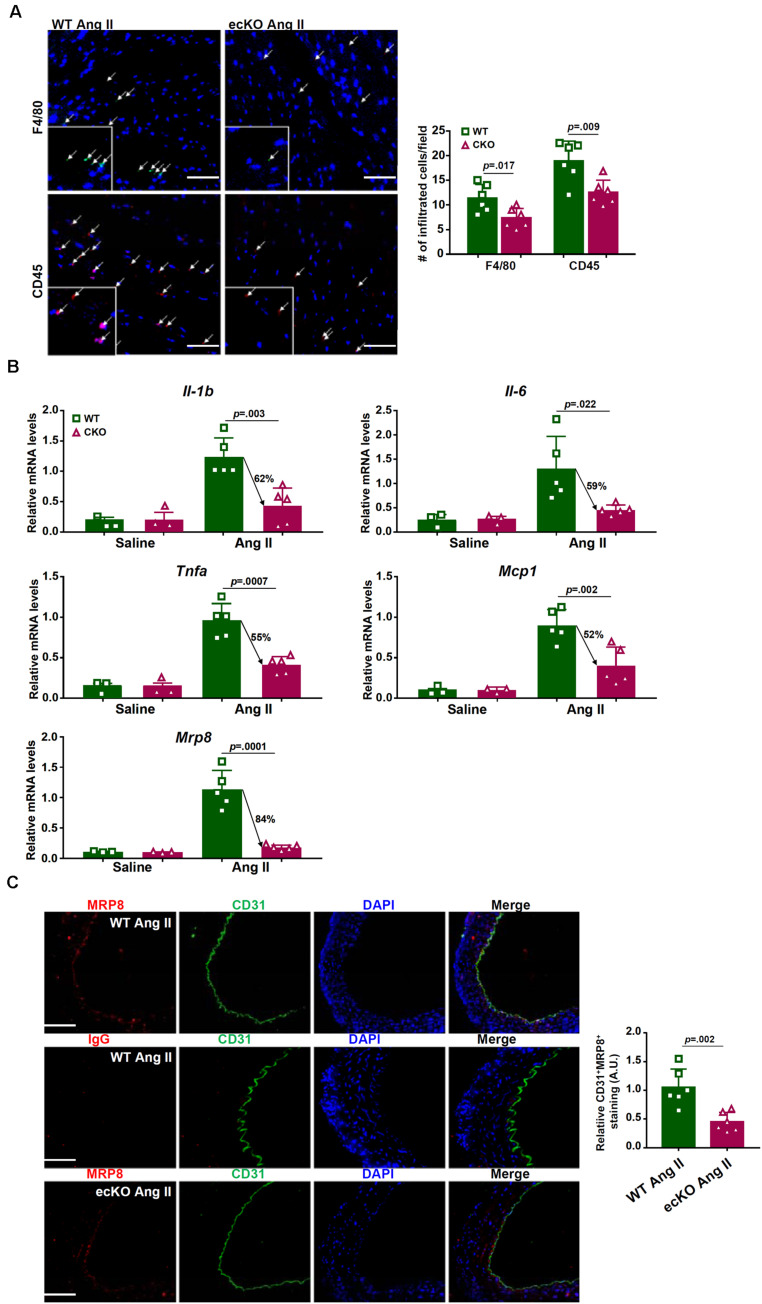
BRG1 regulates endothelium-derived MRP8 in mice. Wild type (*Smarca4*^f/f^) and endothelial BRG1 knockout (*Smarca4*^f/f^; *Cdh5*-Cre) mice were implanted with Ang II minipumps to induce cardiac hypertrophy as described in Methods. **(A)** Cardiac macrophage and leukocyte infiltration were evaluated by immunofluorescence staining with an anti-F4/80 antibody or an anti-CD45 antibody. Stainings were quantified by Image Pro and expressed as relative infiltration of cells. **(B)** Expression levels of pro-inflammatory mediators in the myocardium were examined by qPCR. **(C)** Endothelial expression of MRP8 was evaluated by double immunofluorescence staining with an anti-CD31 antibody and an anti-MRP8 antibody. *N* = 5 mice for each group.

Because macrophage-mediated cardiac inflammation plays a key role in the pathogenesis of pathological hypertrophy, we evaluated macrophage infiltration by immunofluorescence staining with an anti-F4/80 antibody or an anti-CD45 antibody. As shown in Figure 2A, macrophage infiltration was less prominent in the ecKO hearts than in the WT hearts. Consistent with this observation, several pro-inflammatory cytokines associated with macrophage function including interleukin 1 (*Il-1b*), interleukin 6 (*Il-6*), tumor necrosis factor (*Tnfa*), and macrophage chemoattractant protein 1 (M*cp1*) were detected to be present at lower levels in the ecKO hearts than in the WT hearts (Figure 2B). Because inflammation is normally coupled with fibrosis, we examined the expression levels of several pro-fibrogenic marker genes. Indeed, expression levels of *Col1a1*, *Col3a1*, and *Acta2* were collectively down-regulated in the ecKO hearts compared to the WT hearts (Supplementary Figure S1).

MRP8 belongs to the well-documented S100A family of calcium-binding chemokines functioning as a damage associated molecular pattern (DAMP) released under stress conditions to promote macrophage homing and tissue inflammation (Wang S. et al., 2018). Compared to the other chemokines/cytokines, expression of MRP8 was more robustly induced by Ang II infusion and more sensitive to BRG1 deficiency (Figure 2B). We hypothesized that decreased macrophage infiltration in the ecKO hearts could be ascribed to down-regulation of MRP8 expression in the endothelial cells. Double immunofluorescence staining showed that MRP8 expression in the vessels (aortic arteries) was largely restricted to the endothelial layer and that MRP8 expression in endothelial cells, as indicated by CD31^+^MRP8^+^ cells, was significantly lower in the ecKO mice than in the WT mice (Figure 2C).

### BRG1 Mediates Ang II Induced MRP8 Expression in Cultured Vascular Endothelial Cells to Promote Macrophage Migration

**FIGURE 3:**
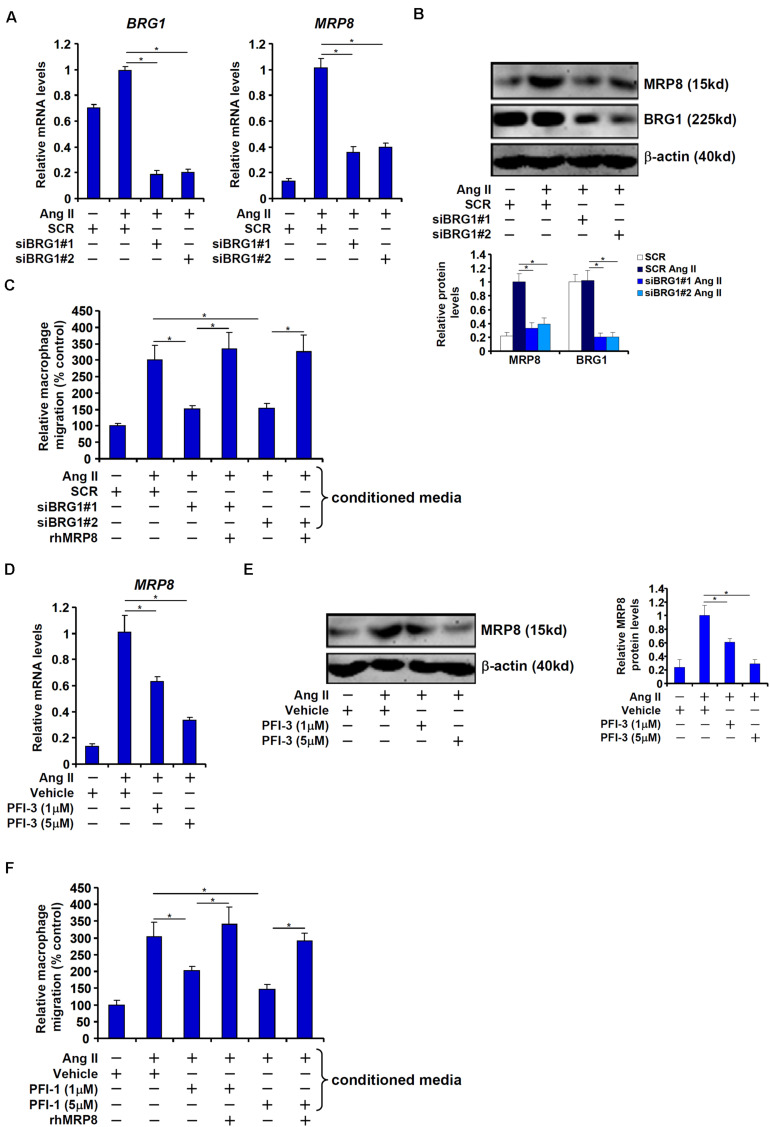
BRG1 mediates Ang II induced MRP8 expression in cultured vascular endothelial cells to promote macrophage migration. **(A–C)** EAhy926 cells were transfected with siRNA targeting BRG1 or scrambled siRNA (SCR) followed by treatment with Ang II (1 μM). MRP8 expression was examined by qPCR and Western. Macrophage migration was examined by transwell assay. **(D–F)** EAhy926 cells were treated with Ang II (1 μM) in the presence or absence of PFI-3. MRP8 expression was examined by qPCR and Western. Macrophage migration was examined by transwell assay. All experiments were repeated three times and one representative experiment is shown. Error bars represent SD (*p < 0.05, one-way ANOVA with *post hoc* Scheffe test).

We next tested the hypothesis that BRG1 mediates Ang II induced MRP8 expression to promote macrophage migration in cultured vascular endothelial cells. Ang II treatment led to robust induction of MRP8 expression in human endothelial cells (EAhy926) at both mRNA (Figure 3A) and protein (Figure 3B) levels; BRG1 knockdown significantly attenuated MRP8 induction by Ang II. In accordance, conditioned media (CM) collected from Ang II-treated endothelial cells displayed much stronger chemoattractive potency than those collected from the mock treated endothelial cells whereas BRG1 depletion dampened the chemoattractive ability of the Ang II treated CM (Figure 3C). Of note, the addition of recombinant human MRP8 to the CM collected from the BRG1 deficient cells restored macrophage migration suggesting that MRP8 may constitute a major Ang II inducible and BRG1-dependent chemotactic substance in the endothelial cells. Similarly, inhibition of BRG1 with a small-molecule compound PFI-3 (Wu et al., 2016) dose-dependently suppressed the induction of MRP8 expression by Ang II in endothelial cells (Figures 3D,E). BRG1 inhibition also crippled the ability of the CM to promote macrophage migration, which could be normalized by the addition of recombinant MRP8 (Figure 3F).

### BRG1 Interacts With HIF-1α to Regulate MRP8 Transcription

**FIGURE 4:**
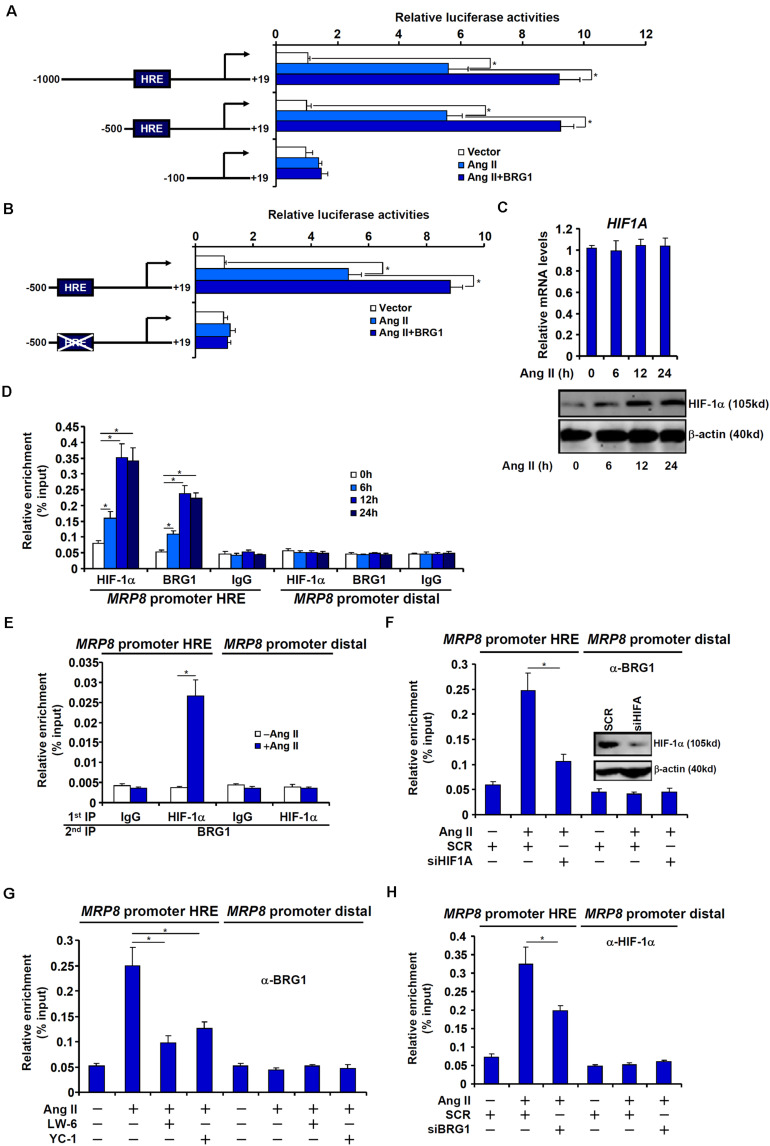
BRG1 interacts with HIF-1α to regulate MRP8 transcription. **(A)** Various MRP8 promoter-luciferase constructs were transfected into EAhy926 cells with or without BRG1 followed by treatment with Ang II (1 μM). Luciferase activities were normalized by protein concentration and GFP fluorescence and expressed as relative luciferase activity compared to the control group. **(B)** Wild type and mutated MRP8 promoter-luciferase constructs were transfected into EAhy926 cells with or without BRG1 followed by treatment with Ang II (1 μM). Luciferase activities were normalized by protein concentration and GFP fluorescence and expressed as relative luciferase activity compared to the control group. **(C,D)** EAhy926 cells were treated with Ang II (1 μM) and harvested at indicated time points. HIF-1α expression levels were examined by qPCR and Western. ChIP assays were performed with anti-HIF-1α, anti-BRG1, or IgG. **(E)** EAhy926 cells were treated with or without Ang II (1 μM) for 24 h. Re-ChIP assay was performed with indicated antibodies. **(F)** EAhy926 cells were transfected with siRNA targeting HIF-1α or scrambled siRNA (SCR) followed by treatment with Ang II (1 μM). ChIP assay was performed with anti-BRG1. **(G)** EAhy926 cells were treated with Ang II (1 μM) in the presence or absence of two different HIF-1α inhibitors. ChIP assay was performed with anti-BRG1. **(H)** EAhy926 cells were transfected with siRNA targeting BRG1 or scrambled siRNA (SCR) followed by treatment with Ang II (1 μM). ChIP assay was performed with anti-HIF-1α. All experiments were repeated three times and one representative experiment is shown. Error bars represent SD (*p < 0.05, one-way ANOVA with *post hoc* Scheffe test).

We next asked whether BRG1 regulated MRP8 expression at the transcriptional level and, if so, by what mechanism. To this end, a series of MRP8 promoter-luciferase constructs with progressively inward deletions were transfected into endothelial cells. Ang II treatment activated the longest MRP8 promoter construct (−1,000 relative to the transcription start site); BRG1 over-expression further enhanced the MRP8 promoter activity (Figure 4A). The responsiveness to Ang II treatment and BRG1 over-expression was retained when the deletion extended to −500 relative to the transcription start site. However, when the deletion went beyond −100, neither Ang II treatment nor BRG1 over-expression was able to activate the MRP promoter activity (Figure 4A). A previous study has identified a DNA binding element for HIF-1α (HRE) between −500 and −100 within the human MRP8 promoter. Mutation of this region abrogated the activation of the MRP8 promoter by Ang II treatment and BRG1 over-expression (Figure 4B), raising the possibility that HIF-1α might be play an essential role in recruiting BRG1. To address this issue, we performed the following experiments. Ang II treatment up-regulated HIF-1α protein, but not mRNA, levels in endothelial cells (Figure 4C). Under normal conditions, HIF-1α protein is modified by prolyl hydroxylases (PHDs) and the ubiquitin E3 ligase VHL before being targeted for degradation. Ang II treatment decreased expression levels of PHD3, but not PHD1 or PHD2 or VHL, which may account for the stabilization of HIF-1α protein (Supplementary Figure S2). More important, Ang II treatment markedly increased the occupancies of the MRP8 promoter by both HIF-1α and BRG1 with similar kinetics (Figure 4D). Ang II-dependent interaction between HIF-1α and BRG1 was further evidenced by Re-ChIP assay (Figure 4E). HIF-1α depletion by small interfering RNA (Figure 4F) or inhibition by two different HIF inhibitors (Figure 4G) equally compromised recruitment of BRG1 to the MRP8 promoter. Of interest, BRG1 deficiency reciprocally influenced the affinity of HIF-1α for the MRP8 promoter as shown by ChIP assay (Figure 4H). Collectively, these data suggest that an interplay between BRG1 and HIF-1α may underscore Ang II induced *trans-*activation of MRP8 in endothelial cells.

### Sequential Recruitment of Histone Modifying Enzymes by BRG1 Contributes to MRP8 *Trans-*Activation

**FIGURE 5:**
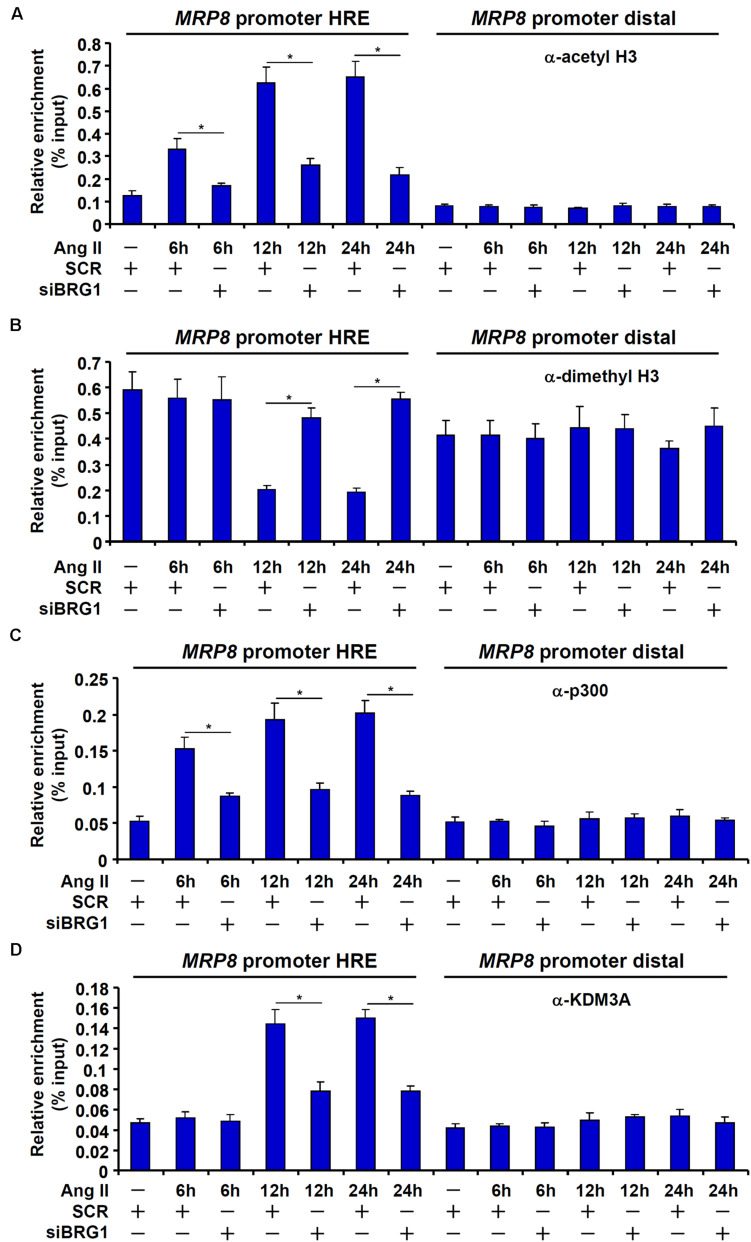
Sequential recruitment of histone modifying enzymes by BRG1. **(A–D)** EAhy926 cells were transfected with siRNA targeting BRG1 or scrambled siRNA (SCR) followed by treatment with Ang II (1 μM). Cells were harvested at indicated time points and ChIP assays were performed with anti-acetyl H3 **(A)**, anti-dimethyl H3K9 **(B)**, anti-p300 **(C)**, or anti-KDM3A **(D)**. All experiments were repeated three times and one representative experiment is shown. Error bars represent SD (*p < 0.05, one-way ANOVA with *post hoc* Scheffe test).

The recruitment of acetyltransferase p300 represents a paradigm of HIF-1α mediated transcription. On the other hand, BRG1 is known to interact with and engage various histone modifying enzymes to regulate transcription. ChIP assays revealed that the MRP8 promoter was characterized by low levels of histone H3 acetylation (Figure 5A) and high levels of dimethyl H3K9 (Figure 5B) indicative of a transcriptionally silenced state. Upon Ang II treatment, H3 acetylation was up-regulated on the MRP8 promoter with a similar kinetics as the recruitment of HIF-1α and BRG1 (Figure 5A). The removal of dimethyl H3K9 from the MRP8 promoter, however, lagged behind the augmentation of H3 acetylation (Figure 5B). Consistent with these observations, it was further discovered that p300 recruitment to the MRP8 promoter synchronized with the binding of HIF-1α/BRG1 whereas KDM3A, a H3K9 demethylase, was brought to the MRP8 promoter with a slightly lagged kinetics (Figures 5C,D).

**FIGURE 6:**
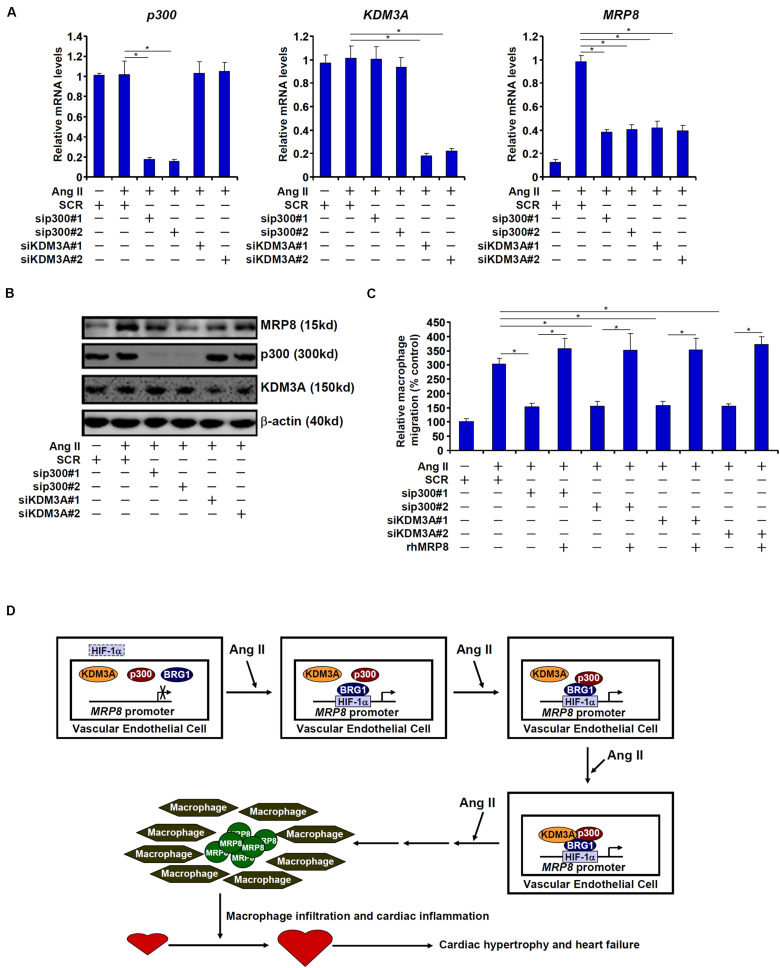
p300 and KDM3A contributes to MRP8 *trans-*activation. **(A–C)** EAhy926 cells were transfected with indicated siRNAs or scrambled siRNA (SCR) followed by treatment with Ang II (1 μM). MRP8 expression was examined by qPCR and Western. Macrophage migration was examined by transwell assay. **(D)** A schematic model.

We finally examined whether endothelial depletion in either p300 or KDM3A would exert equivalent effects on MRP8 expression and macrophage migration. As shown in Figures 6A,B, siRNA-mediated knockdown of p300 and KDM3A comparably down-regulate MRP8 induction by Ang II in endothelial cells. Consequently, conditioned media collected from endothelial cells depleted of either p300 or KDM3A were compromised in the ability to promote macrophage migration (Figure 6C). Combined, these data suggest that sequential recruitment of histone modifying enzymes by BRG1 contributes to MRP8 *trans-*activation.

## Discussion

Macrophage mediated inflammation is considered a key pathogenic factor in a wide range of human diseases (Wynn et al., 2013; Wajant and Siegmund, 2019). Specific chemoattractive cues need to be produced and released to steer the migration of circulating macrophages to host tissues and organs to initiate the inflammatory response (Noels et al., 2019). Transcriptional regulation of these chemokines is orchestrated by the interplay between sequence-specific transcription factors and epigenetic factors. Here we report that the chromatin remodeling protein BRG1 interacts with HIF-1α to activate the transcription of MRP8, a chemoattractive molecule and a damage-associated molecular pattern, in endothelial cells to promote macrophage migration. Moreover, endothelial-conditional BRG1 knockout mice display attenuated macrophage accumulation in the myocardium in a model of cardiac hypertrophy (Figure 6D).

Accumulating evidence argues for a key role for BRG1 in regulating endothelial dysfunction. It is now clear that BRG1 regulates a panel of endothelial derived secreted factors, so-called angiocrine factors, to influence an array of pathophysiological events. BRG1 can potentially activate the transcription of endothelin (ET-1), an endothelial derived vasoconstrictor, promote hypoxia-induced pulmonary hypertension (Yang et al., 2013) and Ang II induced cardiac hypertrophy (Weng et al., 2015). BRG1 has also been demonstrated to activate the transcription of caveolin (CAV1), which in turn suppresses the production of NO and promotes liver fibrosis (Shao et al., 2020). In addition to MRP8 as reported here, several other chemoattractive substances synthesized and released from endothelial cells have been uncovered as transcriptional targets for BRG1. In a model of CaCl_2_ induced abdominal aortic aneurysm, BRG1 mediates TNF-α induced transcription of colony stimulating factor (CSF1) in endothelial cells to stimulate macrophage trafficking (Zhang et al., 2018b). In another model of renal ischemia-reperfusion injury, BRG1 promotes the infiltration of several different lineages of immune cells in the kidneys likely by activating the transcription of endothelial derived IL-33 (Liu et al., 2019a). Still, there is evidence to suggest that BRG1 regulates the transcription of MCP-1 in endothelial cells to stimulate macrophage infiltration in the kidneys in a mouse model of obstructive nephropathy (Liu et al., 2019b). In this regard, it needs to be pointed out that our proposed model (Figure 6D) is unlikely to encompass entirely the observed phenotypes of the ecKO mice exposed to chronic Ang II infusion. Rather, we suspect that BRG1 may dictate the secretome of endothelial cells in response to different stress cues to shape the micro-environment influencing the development and progression of diseases. Single cell based sequencing techniques have recently gained considerable traction in the transcriptional studies (Hu et al., 2018; Nguyen et al., 2018; Xiao and Guo, 2018; Morrish et al., 2019). Exploiting this technology would hopefully help define a genomewide role for BRG1 in endothelial cells in disease models.

We show here the BRG1 activates MRP8 transcription via interaction with HIF-1α. It is reasonable to speculate that mice with endothelial HIF-1α deficiency would phenocopy the BRG1 ecKO mice and develop attenuated cardiac hypertrophy when compared to the control mice. Contrary to this hypothesis, Wei et al. have reported that endothelial deletion of HIF-1α, achieved by the *Tie2*-Cre driver instead of the *Cdh5*-Cre as in our study, leads to aggravated cardiac hypertrophy in mice exposed to pressure overload, which the authors attribute to increased apoptosis of cardiomyocytes (Wei et al., 2012); it remains to be determined whether macrophage infiltration in the myocardium is altered. One possible explanation for this apparent discrepancy is that regulation of MRP8 by HIF-1α does not represent the overarching function of HIF-1α in endothelial cells in the settings of cardiac hypertrophy. Alternatively, it has long been known that the *Tie2*-Cre driver used in the Wei study is not as specific as the *Cdh5*-Cre strain used in the present study (Payne et al., 2018). As frankly admitted by the authors, HIF-1α was deleted by equivalent efficiency in both endothelial cells and cardiomyocytes by this Cre driver thus confounding the data interpretation (Wei et al., 2012). Of intrigue and more in line with our model, Toullec et al. have recently reported that endothelial HIF-1α deletion mediated by the *Cdh5*-Cre driver is protective in a model of enteritis accompanying reduced macrophage aggregation in the intestines (Toullec et al., 2018). Clearly the role of endothelial HIF-1α in the pathogenesis of cardiac hypertrophy deserves further attention.

Our data demonstrate that BRG1 contributes to MRP8 *trans-*activation by sequentially recruiting p300 and KDM3A to the MRP8 promoter. This is consistent with previous reports that BRG1 can interact with histone acetyltransferases (Edelstein et al., 2005; Li et al., 2019a), histone methyltransferases (Shao et al., 2019; Zhang et al., 2019), and histone demethylases (Zhang et al., 2018c) in endothelial cells. The pathophysiological relevance of this finding, however, is unclear. Whereas p300 is sufficient and essential for the induction of cardiac hypertrophy both in cell culture (Yanazume et al., 2003) and in mice (Miyamoto et al., 2006), an endothelial restricted role remains to be explored. Similarly, cardiomyocyte conditional knockout of KDM3A in mice defies the development of pressure overload-induced cardiac hypertrophy (Zhang et al., 2018a); it is not clear whether KDM3A deletion in endothelial cells would recapitulate this phenotype. Future studies harnessing new transgenic mice will hopefully solve these lingering issues.

In summary, our data delineate a novel epigenetic pathway that links the activation of endothelial-derived “alarmin” MRP8 to macrophage migration and cardiac hypertrophy. Because BRG1 is dispensable for the normal endothelial function, targeting endothelial BRG1 might be considered as a safe and effective strategy to combat cardiac hypertrophy and heart failure.

## Data Availability Statement

The raw data supporting the conclusions of this article will be made available by the authors, without undue reservation, to any qualified researcher.

## Ethics Statement

The animal study was reviewed and approved by the Nanjing Medical University Ethics Committee on Humane Treatment of Experimental Animals.

## Author Contributions

XK, TL, and BX conceived the project and provided the funding and supervision. ZL, LY, and YX designed the experiments. ZL, YYZ, LY, and YXZ performed the experiments, collected the data, and analyzed the data. YX wrote the manuscript with inputs from all authors. All authors contributed to the article and approved the submitted version.

## Conflict of Interest

The authors declare that the research was conducted in the absence of any commercial or financial relationships that could be construed as a potential conflict of interest.
