# Corrigendum: BRG1 Stimulates Endothelial Derived Alarmin MRP8 to Promote Macrophage Infiltration in an Animal Model of Cardiac Hypertrophy

**DOI:** 10.3389/fcell.2021.650029

**Published:** 2021-01-26

**Authors:** Zilong Li, Yuanyuan Zhang, Yangxi Zhang, Liming Yu, Bin Xiao, Tianfa Li, Xiaocen Kong, Yong Xu

**Affiliations:** ^1^Pancreas Center, First Affiliated Hospital of Nanjing Medical University, Nanjing, China; ^2^Key Laboratory of Targeted Intervention of Cardiovascular Disease and Collaborative Innovation Center for Cardiovascular Disease, Department of Pathophysiology, Nanjing Medical University, Nanjing, China; ^3^Institute of Biomedical Research, Liaocheng University, Liaocheng, China; ^4^Department of Cardiovascular Medicine, Affiliated Hospital of Hainan Medical University, Haikou, China; ^5^Department of Endocrinology, Nanjing First Hospital, Nanjing Medial University, Nanjing, China

**Keywords:** transcriptional regulation, macrophage infiltration, endothelial cell, Angiotensin II, cardiac hypertrophy

The affiliation for one of the co-corresponding authors (Xiaocen Kong) should be “Department of Endocrinology, Nanjing First Hospital, Nanjing Medial University, Nanjing, China.”

The authors apologize for this error and state that this does not change the scientific conclusions of the article in any way. The original article has been updated.

