# Supplementary material for: BRG1 Stimulates Endothelial Derived Alarmin MRP8 to Promote Macrophage Infiltration in an Animal Model of Cardiac Hypertrophy
Source: Front Cell Dev Biol. 2020 Jul 7;8:569. doi: 10.3389/fcell.2020.00569 (PMC7358314; doi:10.3389/fcell.2020.00569)
Supplement: Supplementary file 1 [file Data_Sheet_1.docx]

**Li ZL et al: BRG1 stimulates endothelial derived alarmin MRP8 to promote macrophage infiltration in an animal model of cardiac hypertrophy**

Online supplementary material

**Fig.S1**: Wild type (*Smarca4*^f/f^) and endothelial BRG1 knockout (*Smarca4*^f/f^; *Cdh5*-Cre) mice were implanted with Ang II minipumps to induce cardiac hypertrophy as described in Methods. Expression levels of pro-fibrogenic genes in the heart were examined by qPCR. N=5 mice for each group.

**Fig.S2:** EAhy926 cells were treated with Ang II (1μM) and harvested at indicated time points. Gene expression levels were examined by qPCR.
